# Effects of implant thread design on primary stability—a comparison between single- and double-threaded implants in an artificial bone model

**DOI:** 10.1186/s40729-020-00239-1

**Published:** 2020-08-20

**Authors:** Yoko Yamaguchi, Makoto Shiota, Masaki Fujii, Masahiro Shimogishi, Motohiro Munakata

**Affiliations:** 1 Department of Implant Dentistry, School of Dentistry, Showa University, 2-1-1 Kitasenzoku Ota-ku, Tokyo, 145-8515 Japan; 2 Oral Implantology and Regenerative Dental Medicine, Department of Masticatory Function Rehabilitation, Division of Oral Health Sciences, Graduate School, Tokyo Medical and Dental University, 1-5-45 Yushima, Bunkyo-ku, Tokyo, 113-8510 Japan

**Keywords:** Primary stability, Single-threaded implant, Double-threaded implant, Artificial bone, Insertion torque, Removal torque, Low-density bone

## Abstract

**Background:**

Primary implant stability is essential for osseointegration. To increase stability without changing the implant size, the thread length must be extended by reducing pitch, using a double-threaded implant, or reducing pitch/lead and lead angle to half that of a single-threaded implant.

**Materials and methods:**

We tested the stabilities of these configurations using artificial bone. A 1.2-mm pitch, single-threaded implant (12S) was the control. We tested a 0.6-mm pitch/1.2-mm-lead double-threaded implant (06D) and a 0.6-mm pitch/lead single-threaded implant (06S). We compared stabilities by measuring insertion torque, removal torque, and the implant stability quotient (ISQ). Damage to bone tissue caused by the implants was evaluated using microscopy and morphometric analysis.

**Results:**

We show that 06D and 06S significantly improved stability compared with the 12S reference. The stability of 06S was significantly greater compared with that of 06D, except for ISQ. The three implants were associated with bone tissue damage characterized by debris and voids surrounding the implant/bone interface. The 06D caused the most tissue damage, followed by 06S and then 12S.

**Conclusion:**

These findings indicate that primary stability was significantly improved by changing the implant size, extending the thread length with reduced pitch/lead, and reducing the lead angle to half that of a single-threaded implant compared with a double-threaded implant.

## Introduction

Secure primary stability is positively associated with successful long-term implant integration to ensure a successful clinical outcome. Initial implant stability is defined as biomechanical stability upon insertion, which is influenced by factors such as bone quantity and quality, geometry of the implant, surgical technique, and insertion torque (IT) [1–4]. New bone develops around the surface of the implant and subsequently undergoes biological fixation (secondary implant stability or osseointegration). Insufficient primary stability is associated with micromotions. After the implant is installed, micromotions > 100 μm may influence osseointegration and bone remodeling by inducing the formation of fibrous tissues and bone resorption at the bone-implant interface [5–7].

Optimal implant design is required for sufficient primary stability [8]. For example, thread design is critically important to achieving primary stability [9, 10]. The relevant characteristics of the thread that determine its functional surface and distribute the biochemical load are as follows: depth, thickness, pitch, and face and lead angles [9]. Certain manufacturers have developed double- or triple-threaded implants [11–13]. Compared with single-threaded implants, multiple-threaded implants can be inserted faster. However, finite element analysis (FEA) revealed that a single-lead thread provides maximum primary stability [14], followed by the double-lead threaded implant [10]. A triple-threaded implant is the least stable [10, 14]. The implant body design can be modified to improve initial stability to increase the success of immediate loading. The thread improves initial stability by maximizing the initial contact area. Further, the thread depth, thread morphology, pitch, and helix angle affect the biomechanical load distribution of the implant [2, 4, 9, 15]. Therefore, commercially available implant systems require better screw designs.

Differences in implant body pitch include an increase in spiral angle with increasing pitch, as represented by multi-threading, and in the pitch itself [9]. To date, however, no studies have used torque or ISQ values that actually reflect the effect of torsion angle and thread compactness on the stability of implants implanted into low bone density bone.

Unfortunately, the effects of double- or triple-threaded implants on primary stability are known for only a few procedures, such as finite element analysis [14]. An excessive lead angle for these implants may jeopardize their ability to sustain axial load despite faster insertion [9]. Further, when micromotion is compared among implants with different lead angles with the same thread pitch, single-threaded implants demonstrate minimal micromotion, whereas triple-threaded implants show maximum micromotion, with both vertical and horizontal loading [14]. Thus, numerous clinicians believe that double-threaded implants can be inserted faster with greater primary stability compared with single-threaded implants. Thus, double- and triple-threaded implants are used for immediate loading of an implant, and the increase in surface area affords greater primary stability. To date, however, data to confirm these findings have been insufficient. We previously conducted a torque analysis to determine the effects of various thread designs on primary stability [16]. However, we were unable to determine the effects of the double-threaded implants.

Further, we employed an artificial bone model to directly observe the implant/bone interface [17]. This method allowed direct observation of the contact interface between an implant and artificial bone without cutting out the test piece. We were able to match the effect of the torque-time curve by grinding the interface of an implant and artificial bones with the aid of a digital microscope.

This model allows observation of immediate bone damage, characterized by debris and voids during implant placement. This in turn provides critical information about the relation between tissue damage and quantifiable factors associated with primary stability, such as torque and implant stability quotient values (ISQ) [9]. Here, we employed this model to measure torque and ISQ, together with direct microscopic observations, to evaluate the primary stabilities of single- and double-threaded implants.

## Methods

### Implants

**Fig. 1:**
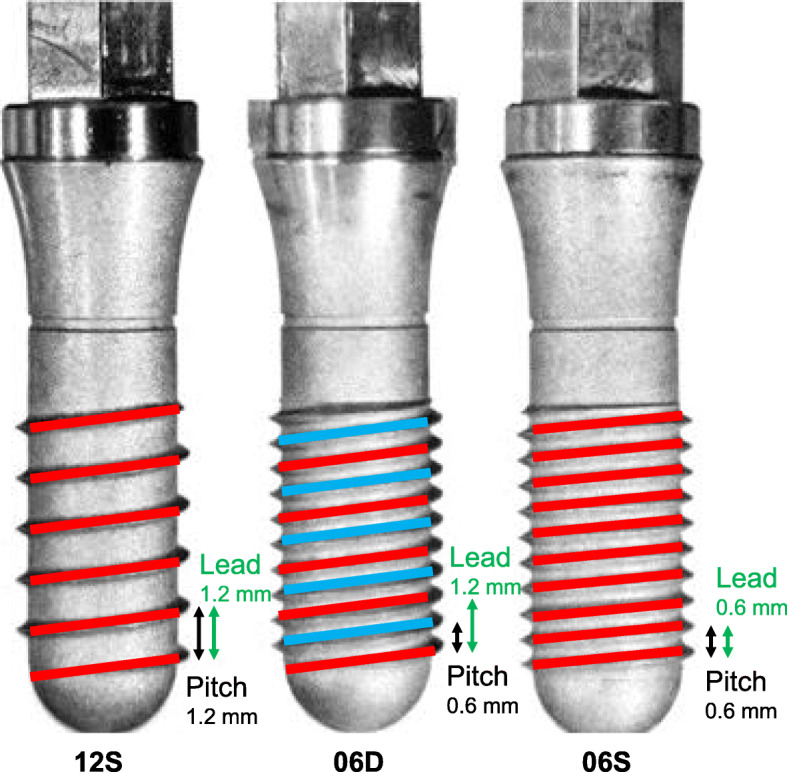
Implants. Implant code 12S is similar to a commercially available standard single-threaded implant with equal thread pitch and a 1.2-mm lead. The thread length of implant code 06D is doubled by adding the second thread (light blue). The thread length of implant code 06S is doubled by a 50% reduction in pitch and lead angles. Characteristics of each implant are summarized in Table 1

Three types of grade-4 titanium cylindrical nonself-tapping implants (codes 12S, 06D, and 06S) were specially designed and manufactured (Suwa Co., Ltd., Fujiyoshida, Yamanashi, Japan) (Fig. 1 and Table 1). The code 12S single-threaded implant served as a reference. Codes 06D and 06S were designed to double the thread length compared to 12S. Code 06D was a double-threated implant with the lead and lead angle equal to those of 12S, although the pitch was reduced twofold because of the second thread. Code 06S was a single-threaded implant, with the pitch, lead, and lead angle reduced twofold compared to those of 12S.

**Table 1:** Dimensions of implants

Implant code	Thread type	Pitch (mm)	Lead (mm)	Lead angle (degree)	Total thread length (mm)
12S	Single-threaded	1.2	1.2	8.1	64
06D	Double-threaded	0.6	1.2	8.1	129
06S	Single-threaded	0.6	0.6	4.65	129

### Artificial bone model

We employed a rigid polyurethane foam (18 cm × 4 cm × 13 cm, Solid Rigid Polyurethane Foam 20 pcf; Sawbones, Vashon, WA, USA) that mimics maxilla molar bone density (0.32 g/cc, similar to that of type 4) and physical properties (compressive strength, 8.4 MPa; tensile strength, 5.6 MPa; shear strength, 4.3 MPa; and coefficient of elasticity, 284 GPa) [18, 19]. The insertion socket was 3.5 mm in diameter and 10.0 mm deep and was prepared using a drill (ASD-360, ASHINA, Hiroshima, Japan).

### Measurement of torque

The implants were inserted with a 500*g* load at 15 rpm. Torque was measured (1 sample/ms) using a PC Torque Analyzer (TRQ-5DRU, Vectrix, Tokyo, Japan). The maximum IT value was acquired when implantation was complete, immediately after which the implant was removed using the same load and rotation speed, and torque was similarly measured. The removal torque (RT) value was acquired at the beginning of the test. Each implant was tested 10 times to achieve sufficient statistical power.

### Assessment of implant stability

Implant stability quotient values (ISQ) were measured using a wireless resonance frequency analyzer (Osstell Mentor, Osstell AB, Gothenburg, Sweden). Each implant was connected to an Osstell SmartPeg (Osstell AB) that transmitted four times at different directions, and 12 measurements were performed using each implant.

### Microscopic examination of contact interfaces

The contact interfaces of the artificial bone (hereafter referred to as “bone”) and implant body were microscopically examined following a published procedure [17]. Briefly, two blocks of artificial bone (2-cm wide, 1-cm deep, and 3-cm high) were assembled into a prismatic column and circumferentially fixated with a metal jig. The insertion socket (3.5-mm diameter, 10.0-mm deep) was introduced into the center of the junction using a drill (ASD-360, ASHINA). The implants were inserted with a 500*g* load at 15 rpm. After insertion terminated, the two bone blocks were simultaneously removed from the metal jags and prismatic column and then separated to observe the contact interfaces. A digital microanalyzer (VHX-1000, Keyence, Osaka, Japan) was used to view the contact interface. Images were morphometrically analyzed using image processing and analysis software (PopImaging, Digital being kids Ltd., Yokohama, Kanagawa, Japan).

### Statistical analysis

Numerical data are presented as the mean ± standard deviation (SD). Data were analyzed using a two-tailed Student *t* test to compare two groups and the Tukey-Kramer method for multiple comparisons (JMP14, SAS Institute Japan, Tokyo, Japan). *P* < 0.05 indicates a significant difference.

## Results

### The IT, RT, and ISQ values revealed significant differences among the implants (Table 2).

**Fig. 2:**
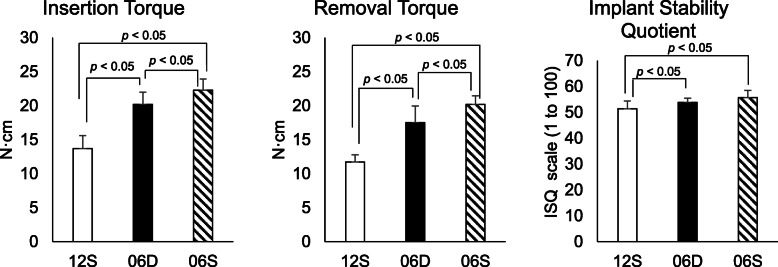
Insertion torque (IT), removal torque (RT), and implant stability quotient (ISQ)

**Fig. 3:**
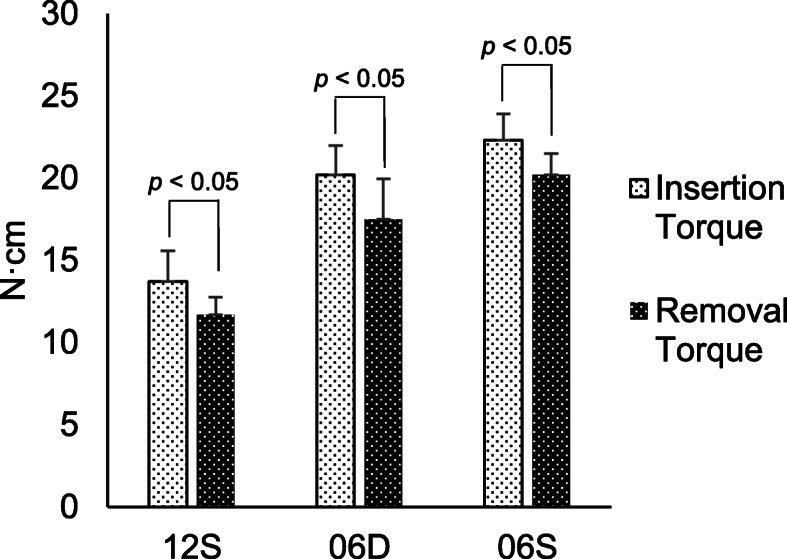
Comparison between IT and RT. Maximum IT value was measured when insertion was terminated. Immediately after insertion, the implant was removed, and the RT value was measured when removal commenced

The IT and RT values of 12S were not significantly different compared with published data (IT, 13.13 ± 1.763 N cm; RT, 12.37 ± 1.746 N cm) (Student *t* test, df = 9, *t* = 2.91, *p* < .017). Compared with 12S, the IT and RT values of 06D and 06S were significantly different (147% and 150%, and 163% and 173%, respectively). The results were analyzed by a statistician who was blinded to the details of the study. The IQ of 06S (22.30 ± 1.68) was significantly higher compared with those of D06 (20.38 ± 1.62), and S12 (13.67 ± 1.78) (Student *t* test, df = 9, *t* = 6.06, *p* < .001) was significantly higher compared with those of D06 (17.45 ± 1.22) (Student *t* test, df = 9, *t* = 5.41, *p* < .001) and S12 (11.69 ± 1.01). The ISQ values of 06D (53.77 ± 2.59) and 06S (55.66 ± 1.55) were significantly higher compared with that of 12S (51.40 ± 2.81). There was no significant difference between 06D and 06S (ANOVA, *F* (2, 27) = 7.24, *p* < .001) (Fig. 2). The RT values of the three implants were significantly lower compared with the IT values (Fig. 3).

**Table 2:** Insertion torque (IT), removal torque (RT), and ISQ values

Code	IT(N•cm)	RT(N•cm)	ISQ
12S	13.67 (1.88)	11.68 (1.06)	51.40 (2.95)
06D	20.19 (1.61)	17.45 (1.28)	53.77 (2.73)
06S	22.30 (1.78)	20.25 (2.47)	55.66(1.62)

### The 12S and 06D implants reached maximum torque twice as fast as the 06S implant

**Fig. 4:**
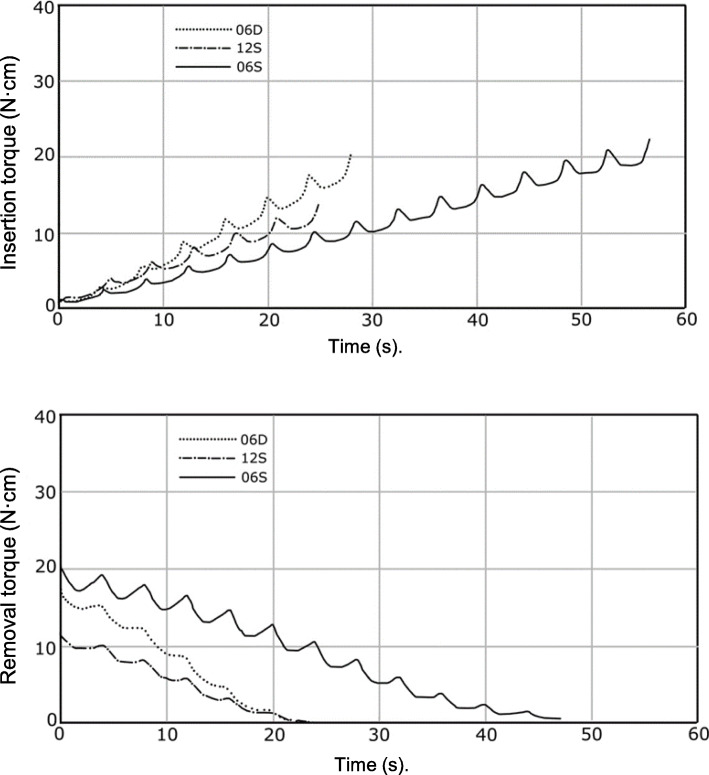
Torque kinetics. Immediately after insertion terminated, the implant was removed using the same load and rotation speed. Torque kinetics were measured during implant insertion (top) and removal (bottom)

The IT values of the three implants increased approximately linearly with time (Fig. 4). The periodicities of the implants were consistent with rotation. Similar maximum torque values were reached by 12S and 06D, which were 2-fold shorter compared with that of 06S, consistent with the lengths of the leads. Maximum removal torque values were reached within 2 s or after a half-turn (Fig. 4). Removal torque-time curves were linear with periodic waves. The removal times were comparable between 12S and 06D, which were 2-fold shorter compared with that of 06S.

### The 06D implant damages bone to a greater extent compared with the 06S or 12S implant

**Fig. 5:**
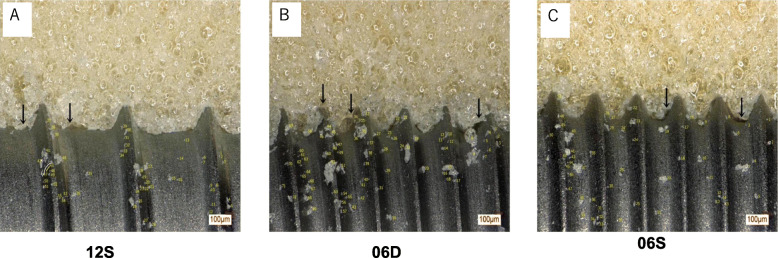
Microscopic analysis of contact interfaces. Microscopic observations of the artificial bone-implant and number of debris particles. The small arrows in the panel indicate voids in the implant-bone interface

**Fig. 6:**
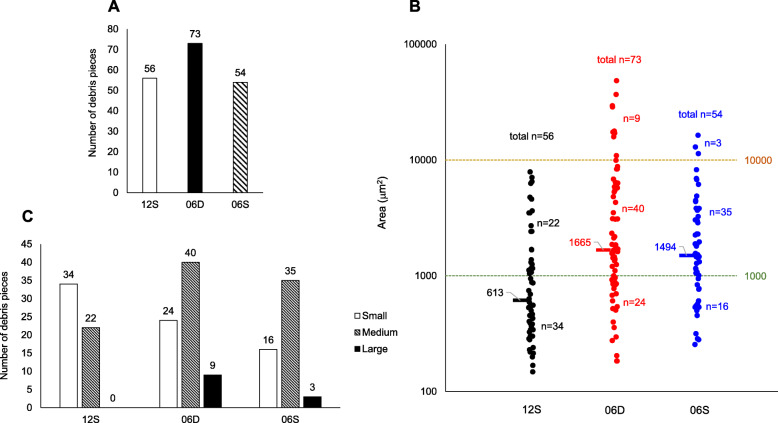
Bone debris at the contact interfaces. **a** Numbers of debris particles. **b** Number and size of debris particles. Each dot indicates a debris particle, and dashes indicate median particle sizes. Particle sizes: small, < 1000 μm^2^; medium, 1000–10000 μm^2^, and large, ≥ 10,000 μm^2^. **c** Particle size distribution

Microscopic observations of the contact interfaces of the artificial bone and implant body revealed minor damage to artificial bone tissue, characterized by debris and voids in the artificial bone that adhered to the implant (Fig. 5a–c). The 06D implant caused the most severe damage, followed by 06S and 12S. In the artificial bone tissue adjacent to 06D, there were numerous voids and abundant debris. Morphometrical analysis revealed that the number of debris particles attached to 06D (73 particles) (Fig. 5b) was higher compared with those of 12S (55 particles) (Fig. 5a) or 06S (52 particles) (Fig. 5c). Although the numbers of debris particles were comparable between 12S and 06S, their sizes were greater in 06S (535.8 μm^2^) compared with those of 12S (305.4 μm^2^) (Fig. 6b). Individual debris particles were classified as follows: small (< 1000 μm^2^), medium (1000–10000 μm^2^), and large (≥ 10000 μm^2^) (Fig. 6a–c). Most debris particles (61%) of 12S were small, with no detectable large debris particles. In contrast, most debris particles associated with 06D and 06S were medium (55% and 65%, respectively) or large (12% and 5.6%, respectively).

## Discussion

We show here that increasing thread length and reducing pitch can increase primary implant stability without changing the size of an implant. Compared with the standard single-threaded implant with a 1.2-mm pitch/lead (12S), torque values and ISQ were significantly increased by doubling the thread length by adding the second thread (06D) or by reducing pitch/lead and lead angle of a single-threaded implant (06S). The torque and ISQ values of 06S were greater compared with those of 06D. Thus, when primary stability must be increased without changing the size of the implant, increasing the thread length and reducing the pitch/lead and lead angle to that of a single-threaded implant is considered more effective versus using a double-threaded implant. Secondary stability occurs in delayed loading and is little affected by thread design. However, multiple threads are chosen when immediate loading is clinically required. Therefore, a single-threaded implant is considered more effective than a double-threaded implant. When immediate loading is required, the failure of the implant may be avoided with the use of single threads. The displacement and micromotion associated with a single-threaded implant is comparable to that of a double-threaded implant with the same pitch and a 2-fold greater lead and lead angle [14]. The torque and ISQ values presented here support the conclusion that 06S outperformed 06D.

Microscopic observations revealed that 06S achieved increased primary stability compared with 06D and explain why the doubled-threaded length of 06D or 06S did not have double the torque value of that of 12S. The three non-self-tapping implants were associated with bone damage, which was more severe using 06D compared with 06S and 12S. We attribute this increased severity of damage using 06D to its shorter pitch compared with that of 12S and larger lead angle compared with those of 12S and 06S. In the artificial bone adjacent to 06D, there were more voids compared with those associated with 06S or 12S, which accounts for the increase in torque. The highest numbers and larger sizes of debris particles were associated with 06D, followed by 0S and 12S, indicating the potential for greater tissue damage. These results likely explain the lower (50%) torque value of 06S compared with those of 06D and 12S.

Although the torque and ISQ values of 06S were greater compared with those of 06D, only the former were statistically significant. These results suggest that although the difference between 06D and 06S in primary stability was small, the risk of slackening was significantly greater for 06D compared with that of 06S. Further, the discrepancy between the statistical significance of the torque and ISQ values may be partly explained by the lower sensitivity of ISQ compared with that of torque. The ISQ values of 06D and 06S compared with that of 12S were 5% and 8% higher, respectively, which were much lower than those of increased torque values. Sakoh et al., who evaluated the primary stability of a conical implant and a hybrid cylindrical screw-type implant according to torque and ISQ values, found that only torque values but not ISQ were significantly different [8]. Resonance frequency analysis (RFA) using the Osstell System and Periotest is commonly used [20–22]. However, neither system optimally measures stability nor defines a successful implant upon implant placement; and measuring IT prevails over the ISQ and Periotest [8, 23].

The IT values reported here ranging from 13 to 22 N cm were much lower than the clinically recommended torque value of 35 N cm [24–26]. The lower torque values can be explained by our use of artificial bone, mimicking type-4 bone, and a cylindrical non-self-tapping design limiting the interface between thread and bone. Moreover, the IT and RT values of 12S were consistent with those of the standard implant (Straumann) measured in our previous study [16].

Here, the RT value of each implant was lower compared with their respective IT values, consistent with other reports [16, 27, 28]. The IT and RT values of 06S were highest, followed by 06D and 12S. In contrast, the differences between RT and IT values were highest for 12S, followed by 06D, and in 06S. We reported that the RT decreased more than IT [8]. Thus, 06S had the lowest rate of decline (IT-RT) in the present study. Therefore, the single thread with a smaller pitch and helical angle is suitable for immediate loading. These inverse results may reflect the relation between lead angle or pitch length and the risk of slackening.

Thread design is a critical factor associated with primary implant stability. FEA loading studies show that relative vertical displacement is affected by thread pitch, torsion angle, and compactness [14]. Under normal load, displacement was positively correlated with thread pitch and helix angle, and negatively with compactness. In low bone density jawbones, implant pitch, helix angle, and compactness have been reported to affect stability. Few studies have clarified the relationship between multiple threads and primary stability [14] [10]. A main advantage of an implant with multiple threads is quicker installation [9]. However, this may be misunderstood by clinicians, because the double-threaded implant recommended by a manufacturer is used for all four types of immediate treatment loading [13]. For example, our comparison of 12S to 06D led us to a different conclusion (i.e., better stabilities of 06D vs 12S and 06S vs 06D). When single- and double-threaded implants with the same pitch were compared, double-threaded implants were less stable because of greater damage to bone tissue damage, which is attributed to the high lead angle reported here as well as by others. Clinicians are advised to recognize the risk associated with using a multithreaded implant with a high lead angle, which may compromise primary stability because of greater bone tissue damage despite faster insertion. The question of placement speed warrants further consideration. Indeed, another advantage of double-threaded implants is placement speed. The implantation speed of 06D was twice that of 06S, and implantation was completed twice as fast. Nevertheless, while plastic bottles and emergency valves have double-threaded screws for faster opening and closing, the effect of placement speed for dental implants on initial stability parameters, such as torque and ISQ values, has not been investigated and remains unknown. It was also interesting that the debris generated with 06D were larger than those generated with 06S, despite higher placement speed. Implant design is the same as that of industrial screws with multiple threads that are mass-produced. This is required because the lead angle is large and the installation speed is fast. Multithreading allows for a lower number of rotations, so even if the installation speed is reduced, the implant is embedded faster compared with a single thread. The issue of placement speed on initial stability warrants further investigation.

Interestingly, not all surfaces of the implant body and implant cavity were in tight contact with each other, and gaps and bone fragments were observed in some portions. Although gaps are unlikely to be beneficial for initial implant stability, bone fragments are thought to be beneficial for bone union [29]. However, it is unclear how the size and number of debris affect bone union. The present study found that different thread designs are associated with different sizes and numbers of debris, but how this affects bone union remains to be addressed.

Of note, artificial bone models simulate physical properties of the real bone, such as density, compressive strength, tensile strength, and elastic modulus, but are distinct in that they have an entirely homogeneous structure. Although bone models composed of a combination of cortical and cancellous bone are also available, this study used a single bone model with a homogeneous density to eliminate any effect of cortical bone and evaluate only the effect of design features on torque and ISQ values. Experimental implant placement using artificial bone models is generally conducted to simulate in vivo implant placement in the jaw bone, and must therefore be designed to provide clear insights into the effects of relevant factors. Regarding observation of the interface between the implant body and the artificial bone using divided blocks, the use of divided blocks is indispensable. To minimize artifacts due to division, the number of divisions should be minimized, and accordingly, two-block division is considered best. Although it is impossible to deny that debris is likely to occur even with two-block division, this is a common error that can occur in all test pieces, and the comparison of implant bodies is therefore considered possible.

Implantation using a bone model is different from in vivo implantation in that implantation is performed under a dry environment, the environmental temperature is not the oral but room temperature, and no physiological reactions occur, such as osteolysis and osteogenesis. Therefore, torque and ISQ values obtained with a bone model cannot be directly extrapolated into in vivo conditions, but can be relatively compared with the corresponding values obtained from other simulation studies under the same conditions. The present study was also conducted for such purposes.

## Conclusion

Despite their advantage of faster insertion, double-threaded implants with a higher lead angle may have diminished primary stability, because they cause increase damage to bone tissue despite faster insertion.

## Data Availability

Not applicable

## Abbreviations

ISQ: Implant stability quotient
IT: Insertion torque
RT: Removal torque
